# Fruit production is influenced by tree size and size‐asymmetric crowding in a wet tropical forest

**DOI:** 10.1002/ece3.4867

**Published:** 2019-01-15

**Authors:** David M. Minor, Richard K. Kobe

**Affiliations:** ^1^ Department of Plant Biology, Program in Ecology, Evolutionary Biology, and Behavior Michigan State University East Lansing Michigan; ^2^ Department of Forestry Michigan State University East Lansing Michigan

**Keywords:** La Selva Biological Station, Costa Rica, multinomial analysis, neighborhood crowding index, ordinal data, plant population and community dynamics, reproduction, resource gradient, soil nutrient availability

## Abstract

In tropical forest communities, seedling recruitment can be limited by the number of fruit produced by adults. Fruit production tends to be highly unequal among trees of the same species, which may be due to environmental factors. We observed fruit production for ~2,000 trees of 17 species across 3 years in a wet tropical forest in Costa Rica. Fruit production was modeled as a function of tree size, nutrient availability, and neighborhood crowding. Following model selection, tree size and neighborhood crowding predicted both the probability of reproduction and the number of fruit produced. Nutrient availability only predicted only the probability of reproduction. In all species, larger trees were more likely to be reproductive and produce more fruit. In addition, number of fruit was strongly negatively related to presence of larger neighboring trees in 13 species; presence of all neighboring trees had a weak‐to‐moderate negative influence on reproductive status in 16 species. Among various metrics of soil nutrient availability, only sum of base cations was positively associated with reproductive status, and for only four species. *Synthesis* Overall, these results suggest that direct influences on fruit production tend to be mediated through tree size and crowding from neighboring trees, rather than soil nutrients. However, we found variation in the effects of neighbors and nutrients among species; mechanistic studies of allocation to fruit production are needed to explain these differences.

## 1 INTRODUCTION

Tree reproduction affects species composition of the forest understory, as well as the future canopy, and can have long‐lasting effects on the forest community. Regeneration is often limited by seed availability in tropical tree species, with the abundance of suitable sites for germination being greater than the number of seeds that reach these sites (De Steven & Wright, 2002; Hubbell et al., 1999; Kobe & Vriesendorp, 2009; Svenning & Wright, 2005). Fruit is a vital food source for frugivore populations in tropical forests, which often have high mortality rates during periods of low fruit production (Fleming, Breitwisch, & Whitesides, 1987; Milton, Giacalone, Wright, & Stockmayer, 2005; Wright, Carrasco, Calderon, & Paton, 1999). Fruit production also represents a major resource investment for trees (Bazzaz, Chiariello, Coley, & Pitelka, 1987; Lord & Westoby, 2006) and may reduce allocation to growth (Charlesworth & Morgan, 1991).

Fruit production at the individual tree level can be influenced by multiple factors, including seed size, tree size, soil nutrient availability, and crowding from neighboring trees. Tree species with large seeds invest more resources in each seed and are likely to produce less fruit (Venable, 1992). Trees must reach a certain developmental stage or size threshold to attain reproductive maturity, although the size associated with reproductive maturity varies among individuals (based on physiology and environment, Owens, 1995) and among species (Thomas, 1996; Wright et al., 2005). After reaching maturity, larger trees are likely to produce more fruit (Greene & Johnson, 1994; Snook, Cámara‐Cabrales, & Kelty, 2005), possibly due to greater access to resources (Carbone et al., 2013; Han, Kabeya, Iio, & Kakubari, 2008). However, even among large, potentially reproductive individuals of the same species, reproduction is unequal, with most of the fruit being produced by a few individuals (González‐Martínez et al., 2006, Herrera & Jovani, 2010; Minor & Kobe, 2017; Moran & Clark, 2012). This variation among individuals indicates that there are additional factors influencing fruit production in trees.

Soil nutrient availability and interactions with neighboring trees may limit fruit production and explain some intraspecific variability in fruit output. Nitrogen (N) and phosphorus (P) fertilization increased reproductive litter in a tropical forest community, but its variation in responses among species was not examined (Kaspari et al., 2008; Wright et al., 2011). Neighboring trees may compete for soil nutrients and cast shade (Baribault & Kobe, 2011; Canham et al., 2006).

A neighborhood crowding index integrates the effects of all between‐individual interactions in the local area surrounding a focal tree, including aboveground and belowground competition, and unmeasured factors incorporating the size, distance, and density of neighbors (Canham, LePage, & Coates, 2004; Coates, Canham, & LePage, 2009; Coomes & Grubb, 2000; Thorpe, Astrup, Trowbridge, & Coates, 2010). Crowding among neighbors may be size‐asymmetric, with larger neighbors being stronger competitors, limiting resources available to smaller trees for reproduction (Wright et al., 2005). Aboveground competition tends to be size‐asymmetric when light availability is a limiting factor (Pretzsch & Biber, 2010), such as in wet tropical forests (Kobe & Vriesendorp, 2011; Record, Kobe, Vriesendorp, & Finley, 2016). Most neighbourhood crowding indices assume size‐asymmetric competition by giving more weight to large neighbors (Biging & Dobbertin, 1992, 1995; Pukkala & Kolström, 1987), and an index can be fully size‐asymmetric by only including neighbors that are larger than the focal tree.

The goal of this study was to investigate how tree size, soil nutrients, and neighborhood crowding influence fruit production in tropical tree species. After testing the assumption that reproductive output will increase with size after reaching a minimum size threshold, we hypothesized that:

H1: Fruit production will increase with nutrient availability.
H2: Fruit production will decrease with local neighborhood crowding from either (a) all neighboring trees or (b) larger neighboring trees.

Our approach was to determine a model which would provide the best prediction of fruit production, and to interpret how these factors influence both the reproductive status and the number of fruit produced. We also used our model output to test whether seed size was negatively related to fruit production in a subset of species.

## 2 MATERIALS AND METHODS

### 2.1 Study site

This study was conducted at La Selva Biological Station, Costa Rica (10°26′N, 84°00′W), a wet tropical forest, receiving approximately 400 cm of precipitation annually (McDade & Hartshorn, 1994). Fruit production measurements and soil samples were taken from five 41 m × 240 m plots which varied in N, P, and base cation availability. The large variation in soil characteristics at La Selva allowed us to sample areas with differing nutrient availability within a relatively small area. Three of these plots are located on older, lower‐fertility, volcanic soils, and two are on younger, richer soils deposited by rivers (McDade & Hartshorn, 1994). All trees 5 cm or greater in diameter at breast height (DBH) within each plot have been mapped and DBH measured approximately annually.

### 2.2 Fruit production measurements

In order to get direct, individual‐level estimates and to avoid potential confounding with animal predation, fruit production was estimated while fruit was still on the tree (Herrera, 1998; LaMontagne & Boutin, 2007; Żywiec, Holeksa, & Ledwoń, 2012). The majority of studies of tree fruit production use litter traps. This approach provides a population‐level estimate of the number of fruit produced, but does not account for losses due to predation and requires modeling of dispersal or incorporating genetic data in order to detect intraspecific variation in reproductive output (Clark et al., 2010; Clark, Ladeau, & Ibanez, 2004; Moran & Clark, 2011). Measurements at the individual level are necessary to understand the factors controlling the amount of fruit produced.

In tropical forests, many trees produce few fruits at any given time, but some trees can have >10,000 fruit in their crowns at once. Counting fruit on individual trees can be time‐consuming and a constraint on sample size. The time consumption could be reduced by explicitly limiting the time spent counting each tree (Koenig & Knops, 2013). An alternative to counting is to place observations into ordinal (i.e., specific ranked order) categories, which is common in social sciences, but rare in ecological studies (Guisan & Harrell, 2000; Podani, 2005). Using ordinal categories instead of full counts can be especially useful for assessing fruit production because the subject of the count is difficult to access and a large range of numbers is expected. To efficiently assess fruit production on a large number of trees, we used ordinal categories instead of full counts.

**Figure 1:**
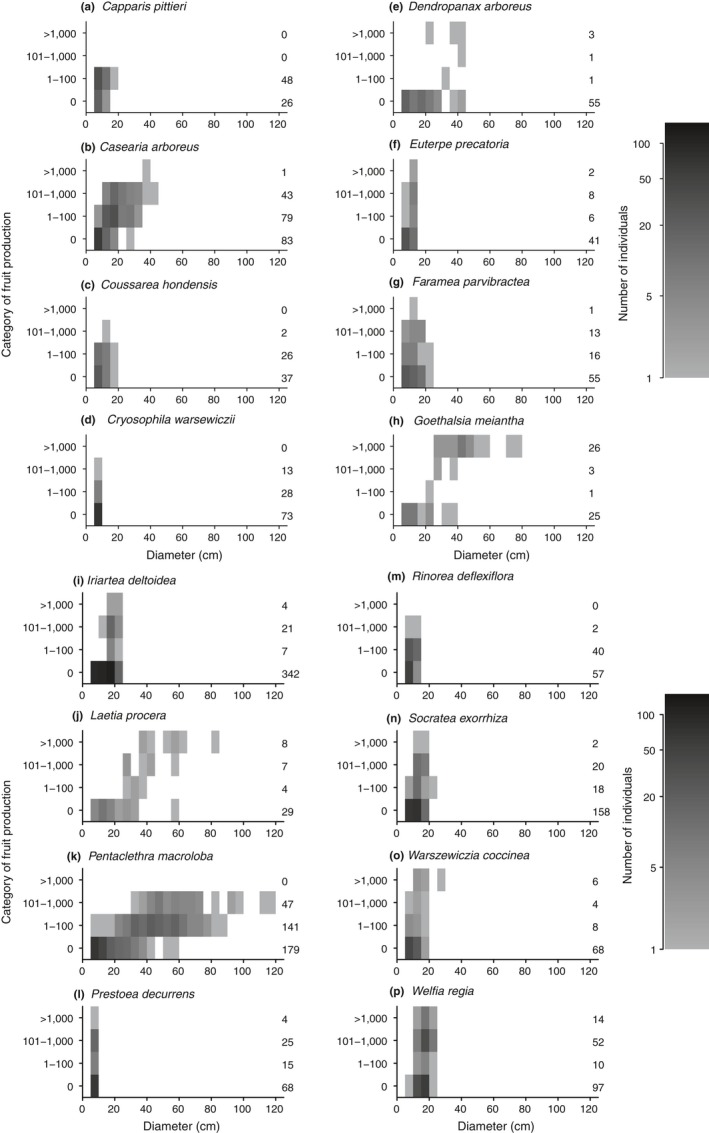
2‐dimensional histogram of fruit production, divided by category and DBH. Number of trees in each fruit production category (vertical axis) for each species divided into 5‐cm DBH classes. Categories are as follows: 1: 0 fruit, 2: 1–100 fruits, 3: 101–1,000 fruits, and 4: >1,000 fruits. The total number of individuals in each category are to the right of each graph. This figure shows the size range for each species, how many individuals were in each fruiting category, and the size distribution for the individuals in each category

**Figure 2:**
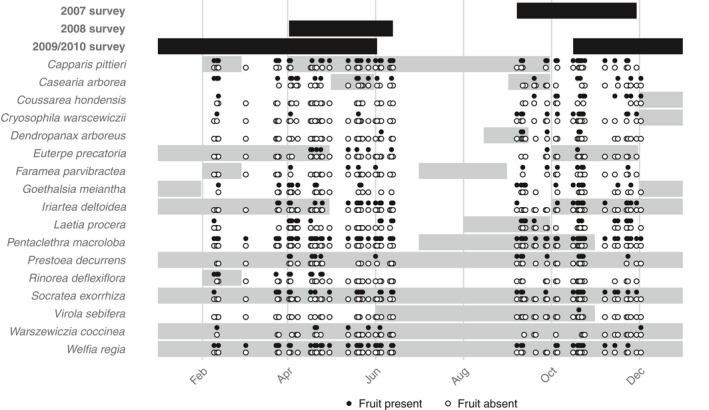
Sampling periods and species phenology. Black bars indicate the time of year of the three sampling periods. Gray bars show the timing of mature fruit, obtained from the literature. When available, phenologies were taken from Frankie et al. (1974). Phenologies for *Coussarea*, *Euterpe*, and *Faramea* were taken from the Digital Flora of La Selva (Vargas & Castro, 2013). Phenology for *Rinorea deflexiflora* was unavailable, so phenology for congener *Rinorea pubipes* is displayed. Points show the dates of all observations: Filled circles indicate cases when fruits were observed, and open circles indicate cases when no fruits were observed

At each plot, fruit production of 17 common canopy and subcanopy tree species (referred to by genus name) was estimated for all mapped individuals. These species were chosen because there were at least 50 individuals of the species present across the five plots, for a total of approximately 2,200 trees (Table 1). For most species, there was good representation across their size range, with some species skewed toward an abundance of smaller individuals (Figure 1). Fruit production was estimated for each individual three times: September–November 2007, April–June 2008, and November 2009–May 2010. These species have diverse fruiting phenologies and inter‐annual cycles of fruit production (Frankie, Baker, & Opler, 1974; Newstrom, Frankie, & Baker, 1994), so the purpose of having three sampling periods at different times of year was to observe each species during its peak fruiting period at least once, rather than to evaluate temporal variability in fruit production across years. Based on published phenological data (Frankie et al., 1974; Vargas & Castro, 2013), the annual fruiting period of every species overlapped with the time of year of at least one of the three sampling periods, although we often observed trees fruiting outside of the published fruiting period (Figure 2). At each observation, the number of fruit was visually estimated and placed into one of four categories:

1. 0 fruit present
2. 1–100 fruits
3. 101–1,000 fruits
4. >1,000 fruits

**Table 1:** Study species at La Selva, Costa Rica. For each species, the number of individuals measured and the number of individuals which were observed fruiting at least once during the study. When available, dry seed mass for each species was obtained from the literature

Species	Family	No. trees	No. observed fruiting	Dry seed mass (mg)
*Capparis pittieri* Standl.	Capparaceae	74	48	721^a^
*Casearia arborea *(L. C. Rich.) Urb.	Salicaceae	206	123	1.32^b^
*Coussarea hondensis* (Standl.) C. M. Taylor & W. C. Burger	Rubiaceae	65	28	128^a^
*Cryosophila warscewiczii* (H. A. Wendl.) Bartlett	Arecaceae	114	41	
*Dendropanax arboreus* (L.) Decne. & Planch.	Araliaceae	60	5	8.0^c^
*Euterpe precatoria* (Mart.) Henderson	Arecaceae	57	16	370^a^
*Faramea parvibractea *Steyerm.	Rubiaceae	85	30	
*Goethalsia meiantha* (Donn. Sm.) Burret	Malvaceae	55	30	4.3^b^
*Iriartea deltoidei* Ruiz & Pav.	Arecaceae	374	32	3,419^b^
*Laetia procera* (Poepp.) Eichl.	Salicaceae	48	19	5.2^b^
*Pentaclethra macroloba* (Willd.) 0. Ktze.	Fabaceae	367	188	3,697^a^
*Prestoea decurrens* (H. Wendl. ex Burret) H. E. Moore	Arecaceae	112	44	167^a^
*Rinorea deflexiflora* H. H. Bartl.	Violaceae	99	42	
*Socratea exorrhiza* (Mart.) H. Wendl.	Arecaceae	198	40	3,421^b^
*Virola sebifera* Aubl.	Myristicaceae	51	1	310.4^c^
*Warszewiczia coccinea* (Vahl) Klotzsch	Rubiaceae	86	18	
*Welfia regia* H. Wendl. ex André	Arecaceae	173	76	1,729^a^
Total		2,173	780	

a McCarthy‐Neumann and Kobe (2008).

b Dupuy and Chazdon (2008)

c Sautu et al. (2006)

We originally included fruit production categories for 1–10 fruits and >10,000 fruits, but observations in these categories were rare so the categories were collapsed into those listed above. Visual estimates were made from the ground using binoculars with 10× magnification.

To assess the accuracy of ordinal categories versus counts, in March 2010 we counted fruit on three reproductive branches on a subset of 675 individuals using a more powerful telescope (20–60× magnification). To estimate total tree fruit, the average of the three branch counts was multiplied by the total number of reproductive branches on the tree. These count estimates were compared to categorical estimates taken within 2 weeks. The majority of individuals were estimated to have zero fruit, but the two estimates agreed for >80% of individuals and were positively correlated (*r* = 0.53 for all comparisons; *r* = 0.71 for 56 comparisons where both estimates were greater than zero), supporting the use of the categorical method. While the Pearson's correlation coefficient (*r*) normally has a maximum of 1, in this case the maximum will be <1 because a continuous variable has been correlated with a categorical variable. A large range of the count estimates corresponds to a single value of the categorical estimates, making the maximum value of the correlation coefficient <1, further strengthening the support for using the categorical method.

### 2.3 Soil resource measurements

Soil samples were taken at a relatively fine spatial scale at each plot. Three subsamples were taken from the upper 20 cm of soil from each meter of a 200‐m transect down the center of the 41 m × 240 m plot and were then composited. In addition, samples were taken in a lattice structure in the rest of the plot with 10‐m spacing between samples (Holste, 2010). Samples were measured for nitrate (${\text{NO}}_{3}^{-}$), ammonium (${\text{NH}}_{4}^{+}$), total extractable phosphorus (P), potassium (K^+^), calcium (Ca^2+^), and magnesium (Mg^2+^). To estimate potential nitrogen mineralization rate (NMin), nitrate and ammonium were also measured after 30‐day incubations. NMin was used in the analyses here because it better represents nitrogen availability over time than ${\text{NO}}_{3}^{-}$or ${\text{NH}}_{4}^{+}$ (Pastor, Aber, McClaugherty, & Melillo, 1984). Also, because K, Ca, and Mg availabilities strongly covaried, they were combined and used in these analyses as the sum of base cation availability (SBC). The final soil nutrient variables included in the models were therefore NMin, P, and SBC. Nutrient availability for each tree was calculated as a distance‐weighted average of the five closest soil sample points, using the R package yaImpute (Crookston & Finley, 2008).

### 2.4 Data analysis

The dataset was analyzed in three ways with different data subsets to test the effects of these factors. First, a binomial logistic regression was used to isolate which factors influence the probability of being reproductive in each species, ignoring variation in the amount of fruit produced. Second, including all individuals, we modeled fruit production in each species with a multinomial logistic regression. Third, because observations of zero fruit were very common (Table 1), a multinomial regression including only reproductive individuals (with fruit observed at least once during the study) was used to determine which of the factors measured are important for the amount of fruit produced following reproductive maturity. Within each of these three regressions, we compared competing covariate sets including tree size, soil nutrients, and neighborhood crowding and evaluated how these factors influenced fruit production within the model. Rather than accepting or rejecting a hypothesis in the classical sense, our strategy was to understand the limitation of each model's applicability for predicting fruit production. These models were fit in a hierarchical Bayesian framework (Clark et al., 2004, 2010), using R and WinBUGS statistical software (Lunn, Thomas, Best, & Spiegelhalter, 2000; R Core Team, 2013).

### 2.5 Neighborhood crowding index

Following Baribault and Kobe (2011) and Canham et al. (2006), the neighborhood crowding index (NCI) was defined as:

(1) $$\text{NCI}=\sum_{i=1}^{n}\left({\text{DBH}}_{i}^{\alpha j}\;\;\exp^{-\nu j/{\text{dist}}_{j}}\right)$$

where *α* and *ν* are random variables controlling the influence of neighbor DBH and distance to focal tree, respectively. Both of these variables were drawn from Gamma (1.0, 1.0) distributions. The effect is summed for *i* = 1, …, *n* neighbors within a 10 m radius of the focal tree. Two sets of neighbors were tested: (a) all trees within 10 m, and (b) within that radius, only individuals that were larger DBH than the focal tree, in order to test for asymmetric neighborhood crowding (ANCI). Neighbors included both conspecific and heterospecific trees. A conspecific neighborhood crowding index was not tested because too few focal trees had conspecific neighbors within 10 m to make meaningful inferences. To keep the number of estimated variables manageable, *α* and *ν* were unique for each focal species *j*, but assumed to be equal for all neighbor species.

### 2.6 Probability of reproduction

We used binomial regression to characterize the probability of reproduction, as a function of tree size, nutrient availability, and neighborhood crowding. If fruit production observations were >0 for an individual, its reproductive status was set to 1. Reproductive status of individual *i* of species *j* (*R_ij_*) was distributed as:

(2) $$R_{\mathrm{ij}}∼\text{Bernoulli}\left(\pi_{\mathrm{ij}}\right)$$

(3) $$\text{logit}\;\pi_{\mathrm{ij}}=X_{j}^{\prime }\beta_{j}+\epsilon_{i}$$

where *π* is the probability of being reproductive, ***X*** *_j_* is the matrix of covariate values for individuals of species *j*, and ***β*** *_j_* is a vector of species‐specific coefficients drawn from:

(4) $$\beta_{j}∼\text{MVNormal}\left(\mu ,\Sigma \right)$$

where *µ* is the mean and Σ is a symmetric, positive definite variance matrix. Individual random effects were included such that: *ϵ_i_* ~ Normal (0.0, *τ*). Random variables *µ*, Σ, and *τ* were drawn from vague prior distributions. Plot‐level random effects were tested in preliminary models. There was no effect of plot, so it was not included explicitly in the model.

Six alternative models were considered for reproductive status (Table 2). Models were fit via the Markov Chain Monte Carlo (MCMC) technique. The models were run for three chains of 50,000 iterations each, following a burn‐in of at least 10,000 iterations to reach convergence (Gelman & Rubin, 1992). Therefore, conclusions were drawn from a posterior distribution of 3 * 50,000 = 150,000 samples. We compared models using deviance information criterion (DIC) and proper scoring rules. DIC is smaller for better fitting models and includes a penalty for additional parameters (Spiegelhalter, Best, Carlin, & Linde, 2002). For an explanation of proper scoring rules, see Supporting Information Appendix S1.

**Table 2:** Model evaluation. Evaluation of the alternative models for the binomial regression (Binom), multinomial regression fit to all individuals (AMulti), and multinomial regression fit to reproductive individuals (RMulti). The binomial regression was a model of the probability of reproduction via observations of fruit presence or absence on individual trees. The multinomial regressions were models of the probability of a tree producing the quantity of fruit in the following categories: 0 fruit; 1–100, 101–1,000, >1,000. RMulti excluded the first category, as only reproductive individuals were included in that model. Alternative covariates include tree diameter (DBH), soil nutrient availability (soil), neighborhood crowding index (NCI), and asymmetric neighborhood crowding index (ANCI). For each model, the deviance information criterion (DIC) is reported, and the lowest DIC for each model is shown in bold. Smaller values of DIC indicate better model fit

Covariates	DIC		
	Binom	AMulti	RMulti
Intercept only	2,419	3,489	993
DBH	1,703	2,749	936
DBH + soil	1,705	2,755	929
DBH + NCI	1,670	2,728	**884**
DBH + ANCI	1,674	**2,718**	900
DBH + soil + NCI	**1,659**	2,738	888
DBH + soil + ANCI	1,679	2,732	910

### 2.7 Quantity of fruit produced

Although the binomial regression can provide useful information about the factors that are associated with reproductive status, it does not reveal how these factors influence the quantity of fruit produced. Because data were collected in ordered categories, we used an ordinal multinomial regression to investigate how tree size, nutrient availability, and neighborhood crowding influenced the number of fruit. Because tree species differ in fruiting phenology, and many tree species reproduce in mast cycles, fruit output, *F_ij_* was defined as the maximum fruiting category observed for each individual *i* of species *j* across all three sampling periods. This model was fit as an ordinal logistic regression such that:

(5) $$F_{\mathrm{ij}}∼\text{Multinomial}\left(\pi_{\mathrm{ijk}}\right)$$

(6) $$\pi_{\mathrm{ijk}}={Q_{\mathrm{ijk}}-Q_{\mathrm{ijk}}}^{-1}$$

(7) $$\text{logit}\;Q_{\mathrm{ijk}}=\gamma_{k}-\left(X_{i}^{\prime }\beta_{i}^{\prime }+\epsilon_{i}\right)$$

where *k* is the fruit number category, *π_k_* is the probability of producing *k* fruit, *Q_k_* is the cumulative probability of producing *k* fruit, and *γ_k_* is the cut point (or boundary) between categories *k* and *k* + 1. A total of *K* = 4 categories were used, as described above, with *K* − 1 ordered cut points such that:

(8) $$\gamma_{1}=0<\gamma_{2}<\gamma_{3}$$

***β*** and *ϵ* were given the same vague priors as in the binomial regression.

The same six alternative sets of covariates were considered for the multinomial regression as in the binomial model (Table 2). Alternative models were compared with DIC and proper scoring rules. The model was run for three chains of 100,000 iterations each, following a burn‐in of at least 10,000 iterations to reach convergence (Gelman & Rubin, 1992). Chains were thinned to every tenth iteration, so conclusions were drawn from a posterior distribution of 3 * 100,000/10 = 30,000 samples.

The same multinomial model was used to examine fruit production in the subset of individuals that were reproductive. The same alternative sets of covariates were used (Table 2). We again compared models using DIC and proper scoring rules (Supporting Information Appendix S1).

### 2.8 Seed mass

We chose not to include seed mass in the models described above because we did not have measurements for all species. However, because seed mass may have an effect on fruit production (Visser et al., 2016), we performed an ancillary analysis to assess how our model results related to seed mass values from the literature (Table 1; Dupuy & Chazdon, 2008; McCarthy‐Neumann & Kobe, 2008; Sautu, Baskin, Baskin, & Condit, 2006). We performed a simple regression between dry seed mass and reproductive threshold size. The reproductive threshold is the diameter at which the probability of reproduction = 0.5, derived from a posterior predictive distribution of the binomial regression. We also performed regressions between dry seed mass and the average probability of each fruit production category across the observed diameter range of a species. Average probability of each fruit production category was derived from a posterior predictive distribution of the multinomial regression fit to all individuals.

## 3 RESULTS

Although fruit production was observed for all 17 species, many individuals did not produce fruit (Table 1). Across all species, fruit production was observed in each fruit production category, with fruit production being more rare in higher categories (Figure 1), which might be attributed to the relative scarcity of larger individuals.

The covariates selected for each of the three analyses are displayed in Table 2. Because these covariate sets were selected for their predictive power, there is no need to further judge their statistical significance. However, in order to differentiate among species, assess the biological significance of each factor, and understand the limitations of each models' predictive power, it is useful to consider the strength of each effect. We have defined strong effects as relationships in which the 95% credible interval (CI) of variable coefficient *β* does not contain zero. Moderate effects are those in which the 87% CI of *β* does not contain zero. All other effects are termed weak effects. 95% and 87% CIs correspond to 2 and 1.5 standard deviations from the mean, respectively.

*Virola* was excluded from analyses because only one individual of this species produced fruit during the study. All three of our sampling periods overlapped with the published fruiting season for *Virola* (June–October, Frankie et al., 1974). It is possible that *Virola* peaks in reproduction in July and August, when we had no observations (Figure 2) or that our plots happened to contain a large proportion of male trees (*Virola* is dioecious, Bawa, Perry, & Beach, 1985, Chazdon, Careaga, Webb, & Vargas, 2003).

### 3.1 Tree size and seed mass

**Figure 3:**
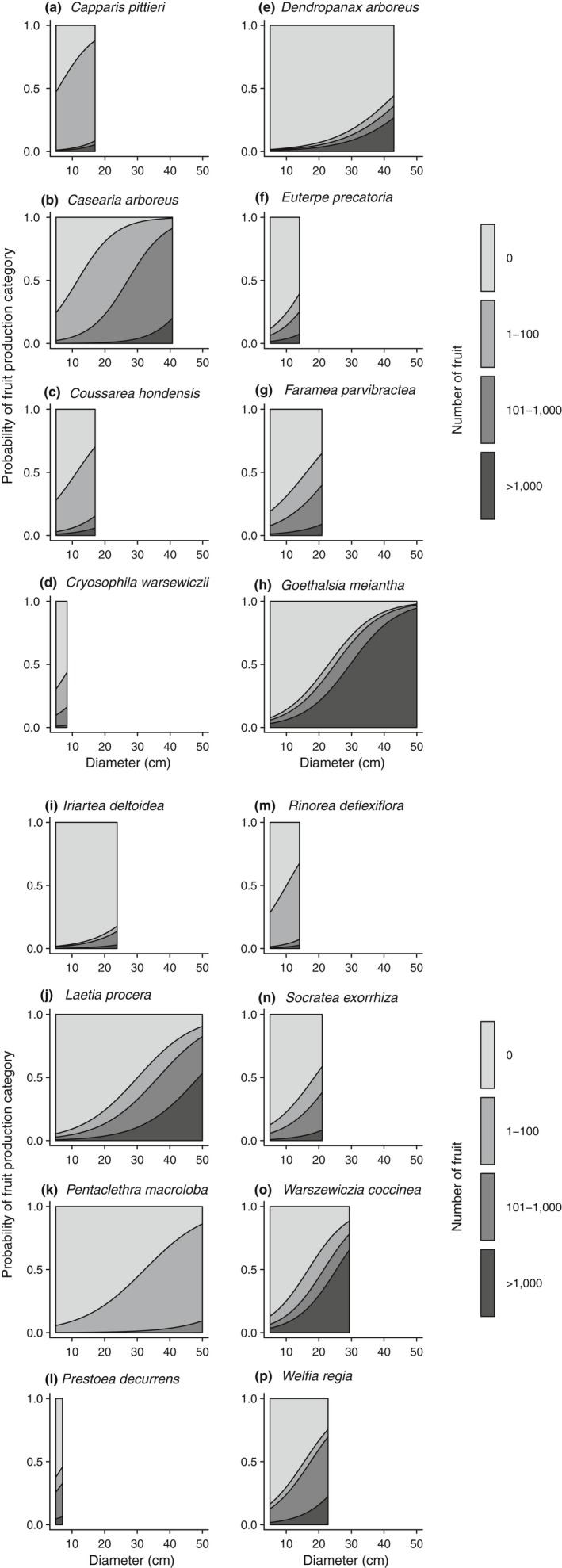
Multinomial relationship between DBH and probability of fruit production for each species. Shaded areas represent the probability of each fruit production category relative to DBH. The probability of each fruit production category at a given DBH is the proportion of the area which is covered by each shade when a line is traced vertically. Darker shaded areas indicate greater fruit production categories

**Figure 4:**
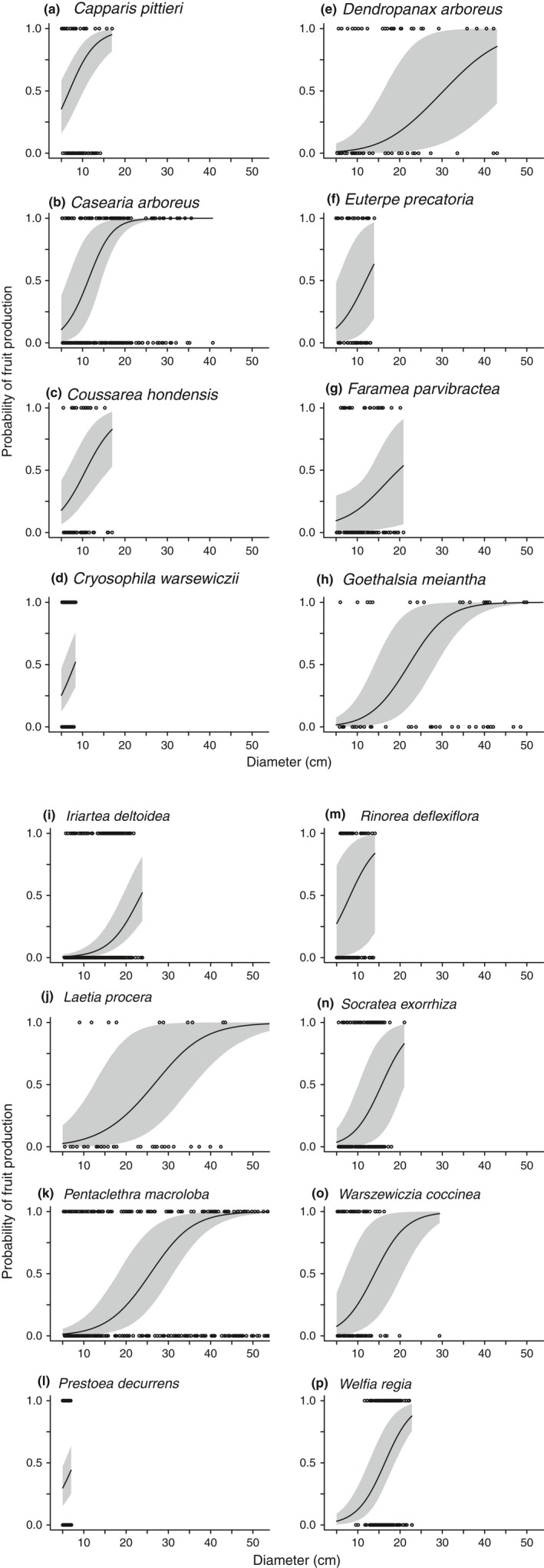
Binomial relationship between DBH and probability of fruit production for each species. Posterior mean probability for individual species is presented with 95% credible intervals indicated with gray shading. Data points are the observed reproductive status of each individual sampled (*r_i_*)

Larger individuals in all species were more likely to be reproductive and produce more fruit (Figures 3, 4). Tree size had a strong effect in every species across all three models, with the exception of *Capparis* in the reproductive‐individual multinomial regression (Supporting Information Tables S4–S6). However, the rate at which fruit production increased varied among species. For example, among the three canopy species *Goethalsia*, *Laetia*, and *Pentaclethra*, we saw a drastic difference in how rapidly fruit production increased with tree size (Figure 3). In *Goethalsia*, moderate levels of fruit production were rare; if an individual was reproductive, it was likely to be capable of producing more than 1,000 fruits. *Laetia* individuals were likely to become reproductive at a similar size as *Goethalsia* individuals, but had a larger size range where moderate levels of fruit production were likely. Finally, even large individuals of *Pentaclethra* produced relatively few fruits.

**Figure 5:**
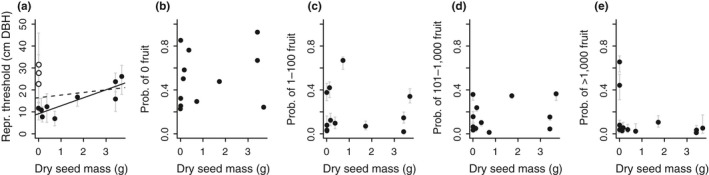
Relationships between seed mass and posterior predictions of fruit production. (a) The reproductive threshold size (probability of fruit production = 0.5) versus dry seed mass for species with seed mass data available from the literature (see Table 1). Each point represents a species. The dotted line shows a linear regression including all species (*p* = 0.5, *R* ^2^ = 0.04), and the solid line shows a linear regression containing only the solid points (*p* < 0.01, *R* ^2^ = 0.74, excluding *Dendropanax*, *Goethalsia*, and *Laetia*). Threshold size was derived from the binomial regression. (b–e) The average probability of each fruit production category (0; 1–100; 101–1,000; >1,000) across the observed size range of each species versus dry seed mass. Linear relationships were not significant. Average probabilities were derived from the all‐individual multinomial regression

*Pentaclethra* does have larger seeds than *Goethalsia* and *Laetia*, but the seed mass did not consistently explain differences in reproduction among species. Seed mass had a positive relationship with reproductive threshold size for a subset of species (*p* < 0.01, *R* ^2^ = 0.74 excluding outliers *Dendropanax*, *Goethalsia*, and *Laetia*; *p* = 0.5, *R* ^2^ = 0.04 with all species). Seed mass did not have a relationship with the average probability of any fruit production category, although the probability of producing >1,000 fruit was low for all species with large seeds (Figure 5).

**Figure 6:**
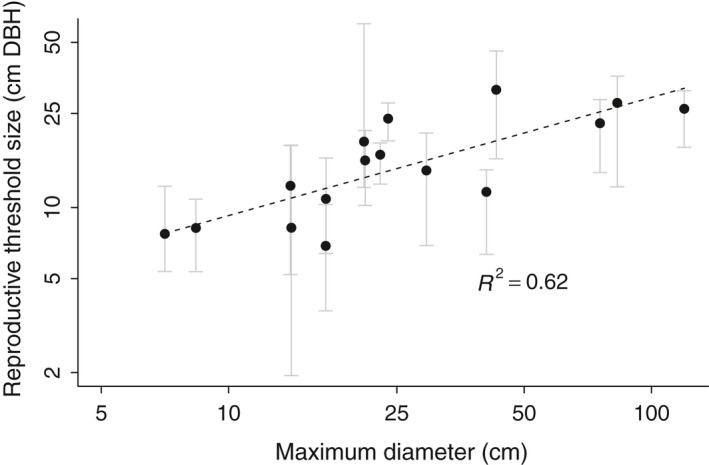
Reproductive threshold size relative to maximum diameter. (a) The reproductive threshold size (*D*_thres_; probability of fruit production = 0.5) versus maximum observed size for each species (*D*_max_). The dotted line shows *D*_thres_ = ½*D*_max_ (*R* ^2^ = 0.62). Threshold size was derived from the binomial regression

Visser et al. (2016) reported an interspecific relationship between the reproductive threshold size (probability of reproduction = 0.5) and the maximum diameter of each species, such that *D*_thres_ = ½*D*_max_ with a coefficient of determination of *R* ^2^ = 0.81. *D*_thres_ is ln(reproductive threshold size) and *D*_max_ is ln(maximum diameter). We tested the same relationship and found *R* ^2^ = 0.62 (Figure 6). The lower coefficient of determination in our case is likely due to having fewer species in our study.

### 3.2 Soil nutrient availability

The sum of base cation availability (SBC) had a strong positive effect on reproductive status in the subcanopy species *Rinorea* and a moderate positive effect in *Capparis*, *Cryosophila*, and *Prestoea* (Supporting Information Table S4), possibly suggesting base cation limitation consistent with H1. The effects of nitrogen mineralization and phosphorus availability were weak and varied in direction.

### 3.3 Neighborhood crowding

**Figure 7:**
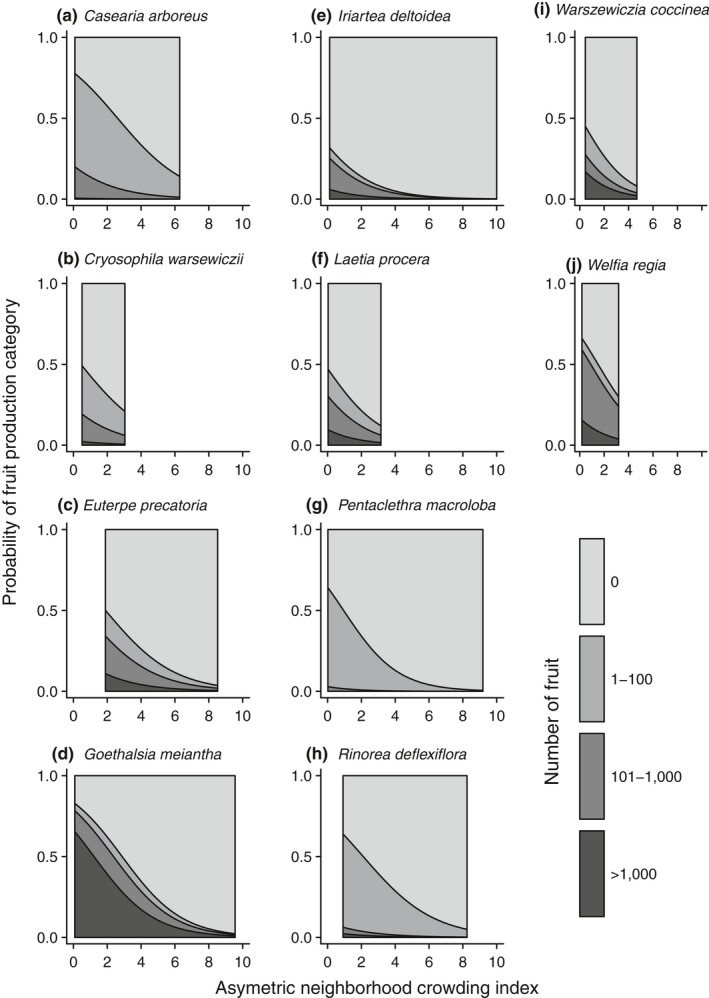
Multinomial relationship between the asymmetric neighborhood crowding index and probability of fruit production for each species. Shaded areas represent the probability of each fruit production category relative to ANCI. The probability of each fruit production category at a given DBH is the proportion of the area which is covered by each shade when a line is traced vertically. Darker shaded areas indicate greater fruit production categories

Neighborhood crowding was selected in some form for inclusion in all three models. NCI was negatively associated with the reproductive status (H2a), with moderate effects in *Rinorea* and *Socratea*, and weak but consistently negative effects in all other species. In the all‐individual multinomial regression, ANCI had a strong negative effect on the number of fruit produced in 13 species (Figure 7) and a moderate negative effect in two species (H2b). The effects of NCI in the reproductive‐individual multinomial regression were weak and varied in direction.

## 4 DISCUSSION

Tree size determined reproductive output in all species. Smaller trees were unable to reproduce until they reached a species‐specific size threshold (Figure 4; Minor & Kobe, 2017; Thomas, 1996; Wright et al., 2005) which was directly related to species maximum size. Our study provided a rare opportunity to confirm that a simple relationship (*D*_thres_ = ½*D*_max_) from Barro Colorado Island, Panama (Visser et al., 2016), holds true at La Selva, Costa Rica (Figure 6). This common finding suggests that this simple relationship may be broadly useful for predicting tree maturation.

Larger trees may have a greater ability to acquire and store nutrients and carbohydrates (Carbone et al., 2013; Greene & Johnson, 1994; Han et al., 2008), and they tended to produce more fruit (Figure 3). Even after accounting for the effect of tree size, reproduction tended to be unequal among conspecifics (González‐Martínez et al., 2006; Herrera & Jovani, 2010; Moran & Clark, 2012), with many individuals not producing fruit at all (Figure 1). Although we found a positive effect of tree diameter for all species, tree height may be a more appropriate size metric in palm species because these species fruit as they grow in height, while growing very little in diameter (Corner, 1966; De Steven, Windsor, Putz, & Leon, 1987).

Competition from neighbors may delay maturation in all species (H2a), and the strong negative association between number of fruit produced and the crowding from larger neighbors (H2b), as opposed to all neighbors, may indicate that competition for light affects reproduction more than competition for soil resources. Carbohydrates which were recently produced from photosynthesis are heavily used in fruit production (Ichie et al., 2013), and decreased light availability decreases fruit production (Greene, Messier, Asselin, & Fortin, 2002; Wright et al., 2005). In contrast, crowding negatively affected growth of individuals in only 3 out of 15 species examined at these same plots (Baribault, Kobe, & Finley, 2012), versus 13 out of 16 species for fruit production. A decrease in fruit production, but not growth, may indicate preferential allocation of carbohydrates to growth in these species. An important caveat is that the crowding index in Baribault et al. (2012) included all neighbors instead of only larger neighbors and a larger neighborhood area. A smaller neighborhood area allowed us to sample trees nearer to the edge of the mapped plots, thus increasing our sample size. A larger sample size was necessary in this study because of how common it was for trees to have zero fruit.

Seed size may explain some variation among species in reproductive threshold size (Figure 5) and number of fruit produced (Visser et al., 2016). Species with larger seeds must allocate more resources to each and so may delay reproduction or produce few seeds at a time. These trade‐offs may also act within a species, with an individual tree producing more, smaller fruit relative to its conspecifics (Primack & Kang, 1989; Venable, 1992), but a tree producing low‐quality seeds could also have a higher rate of abortion (Stephenson, 1981).

Nutrient availability did not affect number of fruit produced, but base cations were positively associated with reproductive status in *Capparis*, *Cryosophila*, *Prestoea*, and *Rinorea*. Base cations may be more limiting for understory species, as these were four of the smallest‐statured species in the study. The lack of more widespread effects of nutrients was surprising, but nutrient availability may affect fruit production indirectly as a consequence of cumulative growth; that is, larger trees grew to that size as a result of higher nutrient availability (e.g. Baribault et al., 2012), and because of their size are able to produce more fruit. Nutrients may also be consumed by costly accessory reproductive structures (structures other than the seed itself), even when seeds are aborted (Lord & Westoby, 2006). Despite little evidence for H1, greater nutrient availability may increase reproduction and seed availability in certain species (Callahan, Fierro, Patterson, & Zafar, 2008; Kaspari et al., 2008; Li, Xu, & Zou, 2006), but temporal variability in fruit production makes this effect difficult to assess (Wright et al., 2011) in the short time period of this study.

The contribution of individuals to population seed availability is highly unequal, with many trees not producing fruit during the study (Figure 1). Even during mast fruiting events, it is common for trees to not produce fruit, even among individuals of reproductive size (Herrera & Jovani, 2010). Lack of reproduction may be due to insufficient nutrient storage by the tree (Han et al., 2008) or a trade‐off with investment in growth (Charlesworth & Morgan, 1991). The 3‐year time span of this study may not have included a mast year for all species. During a mast year, we would expect the peak in fruit production to make resource limitation more apparent than in other years. Without a longer time series of reproductive data, it is difficult to determine whether this study included a peak in fruit production for all species.

Despite accounting for physiological factors (tree size and nutrient availability) and community factors (neighborhood crowding), there was substantial uncertainty in predicting the reproductive status of an individual (Supporting Information Tables S1–S3). In this study, we demonstrated that most common requirements for reproduction across species were large tree size and a lack of competition from larger trees. Species differences in reproductive requirements may influence their reproductive output, affecting seed availability and ultimately future forest composition. More mechanistic studies of allocation to growth versus fruit production across species and biomes will help determine how allocation trade‐offs vary among species.

## CONFLICT OF INTEREST

None declared.

## AUTHOR'S CONTRIBUTIONS

DM and RK conceived the ideas and designed methodology; DM collected the data; DM and RK analyzed the data; DM led the writing of the manuscript. All authors contributed critically to the drafts and gave final approval for publication.

## Data Availability

Data used in this project have been archived at the Knowledge Network for Biocomplexity. urn:uuid:207ba2de‐71bb‐4c7d‐b793‐0232993719f8.
