# Supplementary material for: Fruit production is influenced by tree size and size‐asymmetric crowding in a wet tropical forest
Source: Ecol Evol. 2019 Jan 15;9(3):1458–72. doi: 10.1002/ece3.4867 (PMC6374663; doi:10.1002/ece3.4867)
Supplement: Supplementary file 1 [file ECE3-9-1458-s001.pdf]

Appendix S1 Model Evaluation

We compared models using deviance information criterion (DIC) for both the binomial and multinomial models. DIC is smaller for better fitting models and includes a penalty for additional parameters (Spiegelhalter et al. 2002).

Because we are interested in being able to predict fruit output, we also used proper scoring rules to compare and assess alternative models. Proper scoring rules are used to evaluate the quality of predictions made from probabilistic models. They provide a measure of predictive performance of the model based on observed data and the predictive distribution, $\boldsymbol{\pi} = \{\pi_i, \ldots, \pi_K\}$ (Gneiting and Raftery 2007). The scoring rule function $S(\boldsymbol{\pi}, i)$ was calculated for each individual i in a holdout set. The holdout set consisted of 100 individuals from the full dataset which was used in the binomial and complete multinomial models. A holdout set of 75 individuals was used from the subset of reproductive individuals. It is common to use multiple scoring rules to evaluate a model, each of which incorporates information from the observed data and predictive distribution in a different way. Four scoring rules for categorical variables were used:

$$\text{Zero-one: } S(\pi, i) = \begin{cases} 1 & \text{if } \pi_i = \max\{\pi_1, \ldots, \pi_K\} \\ 0 & \text{if otherwise} \end{cases} \tag{1}$$

$$\text{Quadratic: } S(\pi, i) = 2\pi_i - \sum_{k=1}^{K} \pi_k^2 - 1 \tag{2}$$

$$\text{Spherical: } S(\pi, i) = \frac{\pi_i}{(\sum_{k=1}^{K} \pi_k^2)^{\frac{1}{2}}} \tag{3}$$

$$\text{Logarithmic: } S(\pi, i) = \log \pi_i \tag{4}$$

The zero-one rule (Equation 1) ignores variability in the predictive distribution, returning zero or one for each individual, resulting in the rate at which the correct category was predicted. The logarithmic rule (Equation 4) also uses a limited amount of information, being based only on the probability of the category of which individual i is a part. The quadratic and spherical rules (Equations 2–3) incorporate information from the entire predictive distribution, evaluating the probability of predicting the correct category in relation to the probability of predicting incorrect categories. Higher scores indicate better predictive performance. For the zero-one and spherical rules this means scores closer to 1, but for the quadratic and logarithmic rules (which are negative) this means scores closer to zero. The median and 95% credible intervals were obtained for the posterior mean of each scoring rule.

References

Gneiting, T. and Raftery, A. E. (2007). Strictly Proper Scoring Rules, Prediction, and Estimation. *Journal of the American Statistical Association*, 102(477):359–378.

Spiegelhalter, D. J., Best, N. G., Carlin, B. P., and van der Linde, A. (2002). Bayesian measures of model complexity and fit. *Journal of the Royal Statistical Society: Series B (Statistical Methodology)*, 64(4):583–639.

Table S1: **Model evaluation for the binomial regression.** For each model, DIC and the median and 95% credible intervals of four proper scoring rules are reported. For DIC, smaller values indicate better model fit. For proper scoring rules, larger values indicate better predictive performance of the model.

Covariates	DIC	Zero-one			Quadratic			Spherical			Logarithmic		
Intercept only	2419	0.28	0.30	0.34	-0.98	-0.94	-0.90	0.46	0.47	0.49	-1.31	-1.22	-1.13
DBH	1703	0.14	0.15	0.17	-1.30	-1.24	-1.19	0.30	0.32	0.34	-2.51	-2.21	-2.00
DBH+ soil	1705	0.15	0.21	0.33	-1.27	-1.13	-0.95	0.31	0.37	0.47	-2.47	-2.05	-1.70
DBH + NCI	1670	0.14	0.20	0.33	-1.34	-1.23	-1.01	0.29	0.33	0.45	-3.88	-2.32	-1.91
DBH + ANCI	1674	0.13	0.15	0.18	-1.29	-1.22	-1.16	0.30	0.33	0.35	-2.47	-2.17	-1.94
DBH + soil + NCI	**1659**	0.16	0.21	0.29	-1.37	-1.26	-1.12	0.28	0.33	0.39	-6.27	-2.76	-2.09
DBH + soil + ANCI	1679	0.13	0.16	0.19	-1.29	-1.23	-1.17	0.30	0.32	0.35	-2.53	-2.25	-2.01

Table S2: **Model evaluation for the multinomial regression fit to all individuals.** For each model, DIC and the median and 95% credible intervals of four proper scoring rules are reported. For DIC, smaller values indicate better model fit. For proper scoring rules, larger values indicate better predictive performance of the model.

Covariates	DIC	Zero-one			Quadratic			Spherical			Logarithmic		
Intercept only	3489	0.63	0.68	0.68	-0.45	-0.43	-0.42	0.73	0.74	0.75	-0.79	-0.77	-0.74
DBH	2749	0.74	0.77	0.79	-0.34	-0.32	-0.31	0.80	0.81	0.82	-0.60	-0.58	-0.56
DBH+ soil	2755	0.73	0.76	0.79	-0.35	-0.33	-0.32	0.79	0.80	0.81	-0.63	-0.60	-0.57
DBH + NCI	2728	0.74	0.77	0.79	-0.34	-0.32	-0.31	0.80	0.81	0.82	-0.61	-0.58	-0.55
DBH + ANCI	**2718**	0.75	0.77	0.80	-0.34	-0.33	-0.32	0.80	0.81	0.82	-0.62	-0.60	-0.57
DBH + soil + NCI	2738	0.73	0.76	0.79	-0.36	-0.34	-0.32	0.79	0.80	0.81	-0.63	-0.60	-0.57
DBH + soil + ANCI	2732	0.74	0.78	0.80	-0.35	-0.33	-0.32	0.80	0.81	0.82	-0.63	-0.60	-0.57

Table S3: **Model evaluation for the multinomial regression fit to only reproductive individuals.** For each model, DIC and the median and 95% credible intervals of four proper scoring rules are reported. For DIC, smaller values indicate better model fit. For proper scoring rules, larger values indicate better predictive performance of the model.

Covariates	DIC	Zero-one			Quadratic			Spherical			Logarithmic		
Intercept only	993	0.68	0.71	0.73	-0.42	-0.40	-0.38	0.75	0.77	0.78	-0.67	-0.63	-0.60
DBH	936	0.68	0.72	0.75	-0.40	-0.38	-0.36	0.77	0.78	0.80	-0.64	-0.61	-0.58
DBH+ soil	929	0.68	0.72	0.76	-0.42	-0.39	-0.36	0.76	0.78	0.79	-0.68	-0.63	-0.58
DBH + NCI	**884**	0.65	0.71	0.75	-0.42	-0.39	-0.37	0.75	0.77	0.79	-0.67	-0.62	0.58
DBH + ANCI	900	0.61	0.69	0.75	-0.54	-0.41	-0.37	0.69	0.76	0.79	-1.18	-0.68	-0.60
DBH + soil + NCI	888	0.67	0.72	0.76	-0.42	-0.39	-0.36	0.76	0.78	0.79	-0.67	-0.62	-0.58
DBH + soil + ANCI	910	0.64	0.71	0.75	-0.49	-0.42	-0.37	0.72	0.76	0.79	-0.95	-0.67	-0.60

Table S4: **Coefficient estimates for the DBH + soil + NCI binomial regression.** For each species, standardized coefficient estimates (β) and the 95% credible interval (CI) are displayed. Parameter values are in bold if the 95% CI does not contain zero, and italics if the 87% CI does not contain zero. Because model selection was used, the CI does not confer significance. CI should only be used as a tool for interpreting strength of relationships and differences among species. This model included tree diameter (DBH), nitrogen mineralization rate (Nmin), phosphorous (P), the sum of base cations (SBC) and the neighborhood crowding index (NCI) as predictors of the probability of fruit production. Model DIC: 1659.

	Intercept		DBH		Nmin	
	Estimate	CI	Estimate	CI	Estimate	CI
Capparis pittieri	**18.82**	(9.92, 27.03)	**18.96**	(9.68, 27.08)	0.14	(-1.61, 1.83)
Casearia arborea	**20.05**	(14.50, 26.53)	**22.61**	(16.61, 30.33)	0.58	(-0.89, 2.58)
Coussarea hondensis	**14.56**	(6.59, 21.50)	**16.20**	(7.24, 23.18)	-0.21	(-1.79, 1.17)
Cryosophila warscewiczii	**19.96**	(12.07, 28.52)	**20.71**	(12.55, 29.15)	0.42	(-1.00, 2.10)
Dendropanax arboreus	**6.26**	(0.59, 13.88)	**12.36**	(6.70, 18.46)	-0.91	(-2.93, 0.99)
Euterpe precatoria	**17.88**	(10.36, 27.67)	**19.79**	(11.52, 29.59)	0.04	(-1.59, 1.73)
Faramea parvibractea	*7.18*	(-1.42, 15.12)	**10.30**	(1.03, 18.18)	-1.30	(-3.79, 0.59)
Goethalsia meiantha	**10.02**	(5.97, 16.14)	**15.50**	(10.86, 22.37)	-0.49	(-2.10, 1.08)
Iriartea deltoidea	**9.95**	(5.95, 15.75)	**16.24**	(11.59, 23.67)	-0.40	(-1.86, 1.21)
Laetia procera	**7.19**	(3.01, 13.86)	**12.45**	(7.31, 17.35)	-0.94	(-2.89, 0.82)
Pentaclethra macroloba	**7.53**	(4.93, 10.51)	**12.97**	(10.26, 15.84)	-0.88	(-2.48, 0.68)
Prestoea decurrens	**17.83**	(7.70, 26.43)	**18.40**	(7.91, 27.12)	-0.09	(-1.93, 1.38)
Rinorea deflexiflora	**22.21**	(14.89, 31.88)	**21.88**	(14.23, 31.11)	0.63	(-1.03, 2.48)
Socratea exorrhiza	**16.39**	(10.91, 23.71)	**20.44**	(14.80, 29.45)	0.22	(-1.07, 1.86)
Warszewiczia coccinea	**16.34**	(10.29, 24.59)	**19.02**	(12.97, 26.39)	0.26	(-1.01, 2.17)
Welfia regia	**14.36**	(10.34, 19.74)	**18.70**	(14.31, 25.37)	0.16	(-0.94, 1.53)

	P		SBC		NCI	
	Estimate	CI	Estimate	CI	Estimate	CI
Capparis pittieri	-0.43	(-2.07, 1.33)	*1.05*	(-0.18, 2.47)	-0.54	(-2.64, 0.80)
Casearia arborea	-0.94	(-2.82, 0.45)	0.56	(-0.44, 1.66)	-0.92	(-4.91, 0.38)
Coussarea hondensis	-0.18	(-1.53, 1.60)	0.18	(-1.13, 1.30)	-0.54	(-2.36, 0.72)
Cryosophila warscewiczii	-0.58	(-2.03, 0.88)	*0.91*	(-0.15, 2.06)	-0.67	(-2.89, 0.53)
Dendropanax arboreus	0.14	(-1.72, 2.24)	-1.42	(-3.44, 0.27)	-0.82	(-3.58, 0.88)
Euterpe precatoria	-0.46	(-2.06, 1.08)	0.57	(-0.63, 2.06)	-0.86	(-4.39, 0.26)
Faramea parvibractea	0.40	(-1.43, 2.79)	-0.70	(-2.97, 1.09)	-0.32	(-3.36, 1.77)
Goethalsia meiantha	-0.24	(-1.84, 1.33)	-1.05	(-2.49, 0.18)	-0.83	(-3.57, 0.51)
Iriartea deltoidea	-0.26	(-1.87, 1.30)	-1.25	(-2.59, -0.12)	-1.02	(-4.46, 0.45)
Laetia procera	0.16	(-1.54, 2.08)	-1.22	(-3.04, 0.50)	-0.70	(-3.14, 0.78)
Pentaclethra macroloba	0.06	(-1.46, 1.59)	-1.25	(-2.58, -0.03)	-0.77	(-3.15, 0.65)
Prestoea decurrens	-0.36	(-1.81, 1.38)	*0.91*	(-0.05, 1.89)	-0.59	(-3.15, 0.75)
Rinorea deflexiflora	-0.69	(-2.61, 1.11)	**1.40**	(0.10, 3.00)	*-0.81*	(-3.33, 0.36)
Socratea exorrhiza	-0.82	(-2.68, 0.47)	-0.10	(-1.27, 1.00)	*-0.93*	(-4.37, 0.20)
Warszewiczia coccinea	-0.48	(-1.82, 0.87)	0.13	(-1.19, 1.52)	-0.79	(-2.86, 0.19)
Welfia regia	-0.56	(-1.92, 0.58)	-0.37	(-1.44, 0.61)	-0.89	(-3.43, 0.23)

Table S5: **Coefficient estimates for the DBH + ANCI multinomial regression fit to all individuals.** For each species, standardized coefficient estimates (β) and the 95% credible interval are displayed. Parameter values are in bold if the 95% CI does not contain zero, and italics if the 87% CI does not contain zero. Because model selection was used, the CI does not confer significance. CI should only be used as a tool for interpreting strength of relationships and differences among species. This model included tree diameter (DBH), and the asymmetric neighborhood crowding index (ANCI) as predictors of the number of fruit produced. Estimates of γ represent estimates of cut points between ordinal categories. Model DIC: 2718.

	Intercept		DBH		ANCI	
Species	Estimate	CI	Estimate	CI	Estimate	CI
Capparis pittieri	**10.14**	(5.13, 15.53)	**10.23**	(4.78, 16.26)	*-0.51*	(-1.22, 0.10)
Casearia arboreus	**9.50**	(7.09, 11.81)	**9.79**	(6.34, 13.05)	**-0.53**	(-1.13, -0.15)
Coussarea hondensis	**7.88**	(4.18, 12.03)	**8.70**	(4.56, 13.25)	**-0.58**	(-1.22, -0.17)
Cryosophila warsewiczii	**9.91**	(5.05, 15.21)	**9.92**	(4.72, 15.18)	**-0.57**	(-1.33, -0.13)
Dendropanax arboreus	**2.69**	(0.44, 5.39)	**5.95**	(2.85, 10.60)	*-0.69*	(-1.80, 0.08)
Euterpe precatoria	**10.40**	(5.54, 16.17)	**10.33**	(4.90, 17.23)	**-0.56**	(-1.33, -0.16)
Faramea parvibractea	**5.89**	(2.07, 8.77)	**7.26**	(2.84, 10.47)	**-0.63**	(-1.39, -0.21)
Goethalsia meiantha	**6.61**	(4.37, 10.50)	**8.77**	(5.93, 15.75)	**-0.59**	(-1.27, -0.02)
Iriartea deltoidea	**5.92**	(3.24, 11.35)	**8.09**	(4.92, 15.69)	**-0.62**	(-1.33, -0.22)
Laetia procera	**4.04**	(2.42, 6.09)	**6.68**	(4.29, 10.29)	**-0.66**	(-1.56, -0.09)
Pentaclethra macroloba	**3.90**	(3.13, 4.74)	**5.84**	(3.84, 7.54)	**-0.71**	(-1.73, -0.20)
Prestoea decurrens	**9.64**	(4.8, 17.64)	**9.97**	(4.90, 17.66)	**-0.56**	(-1.4, -0.06)
Rinorea deflexiflora	**11.16**	(6.34, 16.72)	**10.68**	(5.20, 16.88)	**-0.55**	(-1.37, -0.12)
Socratea exorrhiza	**8.27**	(4.00, 19.58)	**10.40**	(5.81, 24.43)	-0.48	(-1.24, 0.61)
Warszewiczia coccinea	**8.79**	(4.72, 14.30)	**9.81**	(6.01, 16.79)	**-0.57**	(-1.24, -0.10)
Welfia regia	**8.49**	(5.35, 15.94)	**9.99**	(6.35, 20.17)	**-0.53**	(-1.11, -0.04)

	γ_2		γ_3	
Species	Estimate	CI	Estimate	CI
Capparis pittieri	4.45	(3.09, 6.33)	4.95	(3.38, 7.17)
Casearia arboreus	2.64	(2.13, 3.21)	6.41	(5.13, 7.99)
Coussarea hondensis	2.60	(1.68, 3.77)	3.71	(2.33, 5.61)
Cryosophila warsewiczii	1.41	(0.96, 1.95)	3.82	(2.53, 5.64)
Dendropanax arboreus	0.40	(0.05, 1.10)	0.84	(0.23, 1.79)
Euterpe precatoria	0.69	(0.29, 1.25)	2.14	(1.23, 3.31)
Faramea parvibractea	1.05	(0.63, 1.57)	3.02	(1.97, 4.44)
Goethalsia meiantha	0.34	(0.04, 0.93)	0.98	(0.37, 1.86)
Iriartea deltoidea	0.33	(0.14, 0.59)	2.03	(1.32, 2.91)
Laetia procera	0.76	(0.26, 1.48)	2.17	(1.23, 3.35)
Pentaclethra macroloba	4.12	(3.40, 4.93)	9.28	(7.14, 11.93)
Prestoea decurrens	0.56	(0.31, 0.87)	2.55	(1.81, 3.48)
Rinorea deflexiflora	3.32	(2.35, 4.54)	4.41	(3.01, 6.33)
Socratea exorrhiza	0.85	(0.52, 1.25)	2.80	(1.90, 3.96)
Warszewiczia coccinea	0.79	(0.37, 1.36)	1.42	(0.79, 2.23)
Welfia regia	0.31	(0.16, 0.51)	2.38	(1.84, 2.98)

Table S6: **Coefficient estimates for the DBH + NCI multinomial regression fit to only reproductive individuals.** For each species, standardized coefficient estimates (β) and the 95% credible interval are displayed. Parameter values are in bold if the 95% CI does not contain zero, and italics if the 87% CI does not contain zero. Because model selection was used, the CI does not confer significance. CI should only be used as a tool for interpreting strength of relationships and differences among species. This model included tree diameter (DBH), and the neighborhood crowding index (NCI) as predictors of the number of fruit produced. Estimates of γ represent estimates of cut points between ordinal categories. Model DIC: 884.

	Intercept		DBH		NCI	
Species	Estimate	CI	Estimate	CI	Estimate	CI
Capparis pittieri	-2.07	(-7.30, 1.45)	2.50	(-1.86, 4.59)	0.69	(-0.87, 3.07)
Casearia arboreus	0.64	(-2.97, 3.29)	**3.89**	(1.64, 5.33)	0.45	(-0.35, 1.36)
Coussarea hondensis	1.59	(-2.12, 6.08)	**4.33**	(1.03, 6.73)	0.25	(-0.49, 1.02)
Cryosophila warsewiczii	**4.17**	(1.34, 7.73)	**5.55**	(3.94, 8.75)	0.16	(-0.27, 0.64)
Dendropanax arboreus	**3.67**	(0.81, 7.10)	**5.29**	(3.70, 7.76)	0.14	(-0.74, 0.67)
Euterpe precatoria	**5.14**	(1.66, 9.25)	**6.08**	(4.24, 9.76)	0.14	(-0.33, 0.91)
Faramea parvibractea	**4.85**	(2.25, 8.31)	**5.91**	(4.33, 9.04)	0.11	(-0.40, 0.56)
Goethalsia meiantha	**5.93**	(3.43, 10.21)	**6.40**	(4.47, 9.21)	-0.04	(-0.95, 0.49)
Iriartea deltoidea	**5.39**	(2.50, 7.82)	**6.09**	(4.45, 7.85)	0.04	(-0.81, 0.64)
Laetia procera	**3.02**	(0.80, 4.88)	**5.00**	(3.69, 6.76)	0.25	(-0.30, 0.90)
Pentaclethra macroloba	**-0.84**	(-1.66, -0.29)	**3.17**	(1.70, 4.20)	0.56	(-0.69, 2.08)
Prestoea decurrens	**5.01**	(0.64, 8.46)	**5.87**	(3.40, 9.15)	0.19	(-0.33, 0.86)
Rinorea deflexiflora	1.10	(-3.32, 6.43)	**4.09**	(0.79, 6.77)	0.28	(-0.93, 1.49)
Socratea exorrhiza	**6.07**	(2.90, 12.06)	**6.55**	(4.42, 11.58)	-0.06	(-0.73, 0.38)
Warszewiczia coccinea	*4.63*	(-1.93, 8.41)	**5.74**	(1.87, 8.98)	0.14	(-0.54, 1.25)
Welfia regia	**6.91**	(3.95, 9.58)	**6.90**	(4.54, 9.98)	-0.11	(-1.13, 0.41)

	γ_2	
Species	Estimate	CI
Capparis pittieri	0.50	(0.01, 1.83)
Casearia arboreus	3.47	(2.39, 4.90)
Coussarea hondensis	1.11	(0.22, 2.74)
Cryosophila warsewiczii	2.61	(1.37, 4.42)
Dendropanax arboreus	0.65	(0.08, 1.76)
Euterpe precatoria	1.70	(0.77, 2.96)
Faramea parvibractea	2.32	(1.26, 3.78)
Goethalsia meiantha	1.05	(0.30, 2.27)
Iriartea deltoidea	2.76	(1.80, 3.94)
Laetia procera	1.80	(0.83, 3.07)
Pentaclethra macroloba	3.89	(2.54, 5.77)
Prestoea decurrens	2.71	(1.78, 3.84)
Rinorea deflexiflora	1.10	(0.23, 2.69)
Socratea exorrhiza	2.58	(1.60, 3.79)
Warszewiczia coccinea	0.86	(0.30, 1.68)
Welfia regia	3.06	(2.34, 3.87)
